# Experiences With Internet Triaging of 9498 Outpatients Daily at the Largest Public Hospital in Taiwan During the COVID-19 Pandemic: Observational Study

**DOI:** 10.2196/20994

**Published:** 2021-07-27

**Authors:** Ding-Heng Lu, Chia-An Hsu, Eunice J Yuan, Jun-Jeng Fen, Chung-Yuan Lee, Jin-Lain Ming, Tzeng-Ji Chen, Wui-Chiang Lee, Shih-Ann Chen

**Affiliations:** 1 Department of Medical Education Taipei Veterans General Hospital Taipei Taiwan; 2 Information Management Office Taipei Veterans General Hospital Taipei Taiwan; 3 Department of Nursing Taipei Veterans General Hospital Taipei Taiwan; 4 Department of Medical Affairs and Planning Taipei Veterans General Hospital Taipei Taiwan; 5 Big Data Center Department of Medical Research Taipei Veterans General Hospital Taipei Taiwan; 6 Institute of Hospital and Health Care Administration School of Medicine National Yang-Ming University Taipei Taiwan; 7 Department of Family Medicine Taipei Veterans General Hospital Taipei, Taiwan, ROC Taipei Taiwan; 8 Division of Cardiology Department of Medicine Taipei Veterans General Hospital Taipei Taiwan; 9 Institute of Clinical Medicine Cardiovascular Research Center National Yang-Ming University Taipei Taiwan; 10 Cardiovascular Center Taichung Veterans General Hospital Taichung Taiwan

**Keywords:** COVID-19, hospital, information services, outpatients, patient, SARS-CoV-2, triage, virus

## Abstract

**Background:**

During pandemics, acquiring outpatients’ travel, occupation, contact, and cluster histories is one of the most important measures in assessing the disease risk among incoming patients. Previous means of acquiring this information in the examination room have been insufficient in preventing disease spread.

**Objective:**

This study aimed to demonstrate the deployment of an automatic system to triage outpatients over the internet.

**Methods:**

An automatic system was incorporated in the existing web-based appointment system of the hospital and deployed along with its on-site counterpart. Automatic queries to the virtual private network travel and contact history database with each patient’s national ID number were made for each attempt to acquire the patient’s travel and contact histories. Patients with relevant histories were denied registration or entry. Text messages were sent to patients without a relevant history for an expedited route of entry if applicable.

**Results:**

A total of 127,857 visits were recorded. Among all visits, 91,195 were registered on the internet. In total, 71,816 of them generated text messages for an expedited route of entry. Furthermore, 65 patients had relevant histories, as revealed by the virtual private network database, and were denied registration or entry.

**Conclusions:**

An automatic triage system to acquire outpatients’ relevant travel and contact histories was deployed rapidly in one of the largest academic medical centers in Taiwan. The updated system successfully denied patients with relevant travel or contact histories entry to the hospital, thus preventing long lines outside the hospital. Further efforts could be made to integrate the system with the electronic medical record system.

## Introduction

Since the outbreak of COVID-19, millions of individuals have been infected and tens of thousands of deaths have been reported worldwide [1]. This highly contagious and virulent disease has a higher case fatality rate than influenza. Moreover, sequelae such as pulmonary fibrosis have been observed in some recovered patients. As such, blocking the spread of the disease is a top priority during the pandemic [2]. Accordingly, governments worldwide have implemented various policies in hopes of decreasing the spread of COVID-19 [3], and the government of Taiwan is no exception [4].

Rigorous measures including travel restrictions and social distancing have been implemented to decrease the risk of community spread [5,6]. Various policies have also been enforced in hospitals to lower the risk of nosocomial infection. To contain the spread of the disease in inpatient departments, visitors to patients have been restricted to specific time slots, and the total number of visitors has been regulated [7,8]. However, while such regulations can be easily executed in an inpatient department, an outpatient department presents a greater challenge owing to the large number of daily outpatient visits and because the screening of ambulatory patients for relevant travel, occupation, contact, and cluster histories is necessary to minimize disease spread in an outpatient department.

Traditionally, screening of the travel, occupation, contact, and cluster histories of outpatients was conducted in examination rooms by physicians. However, given the current circumstances, allowing outpatients with a history of travel to high-risk countries to enter a hospital poses a risk of disease spread in the ambulatory service department [9]. A system to efficiently screen outpatients with relevant histories and deny them entry to the hospital is therefore of utmost importance. Thus, an automatic system that can acquire each patient’s relevant travel and contact histories was deployed rapidly in Taipei Veterans General Hospital, one of the largest academic medical centers in Taiwan.

The aim of this study was to illustrate the deployment and utilization of the system. Further analysis focused on dynamic changes in the daily ambulatory services of the medical center with the implementation of this system. The experiences reported here will likely help hospitals worldwide in combating the COVID-19 pandemic and future pandemics.

## Methods

### Methods Overview

Taipei Veterans General Hospital is the largest public academic medical center in Taiwan. As of March 2020, this hospital had 2800 beds served by a staff of 6670 members. Until then, >8000 patient visits have been recorded on a daily basis. To contain the spread of COVID-19 in the outpatient department, the hospital deployed an automatic system to acquire patients’ relevant travel and contact histories.

### System Design

**Figure 1:**
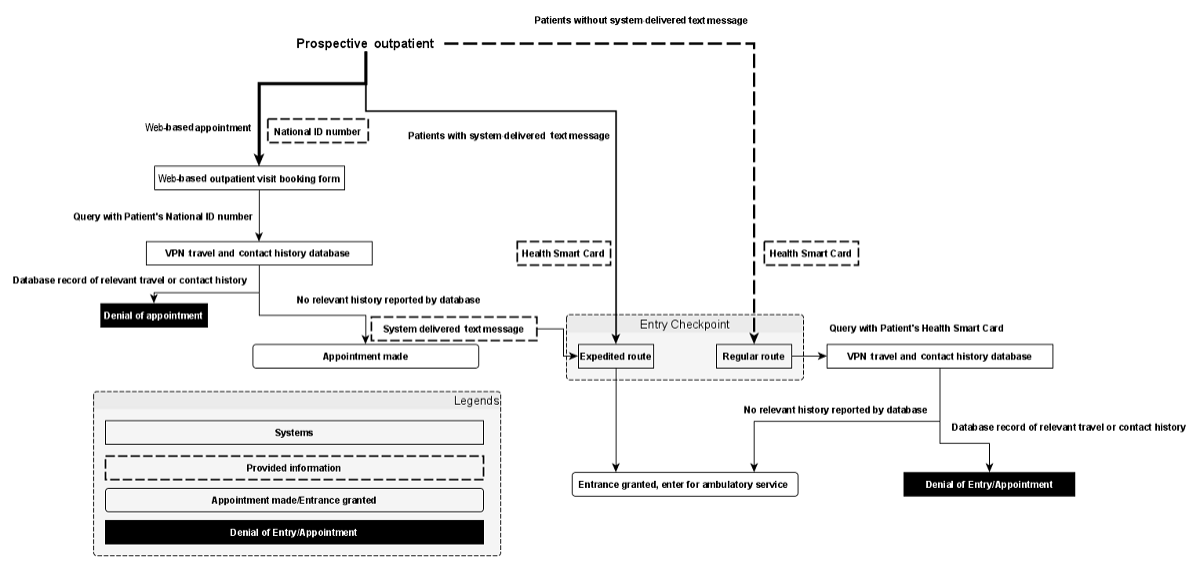
Design of the outpatient screening system. VPN: virtual private network.

**Figure 2:**
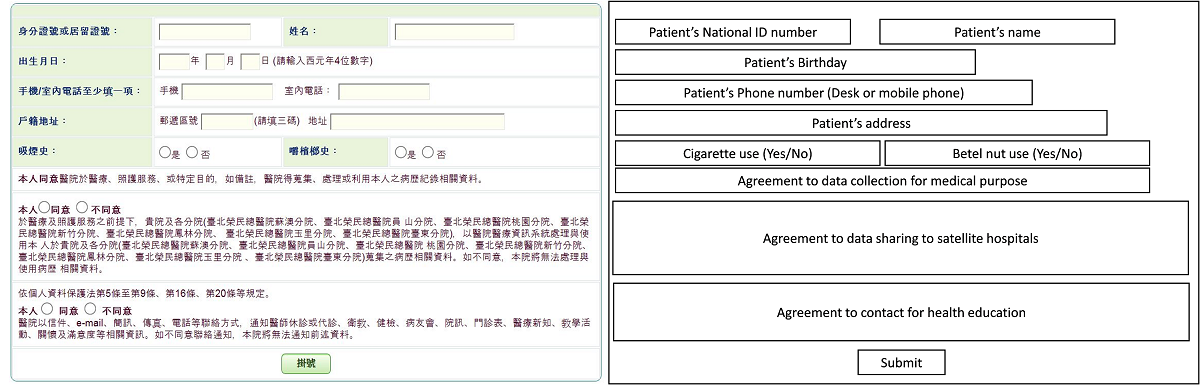
Screenshot of a web-based appointment form.

A web-based appointment system for the hospital’s ambulatory service was already in operation before the incorporation of the screening system. The system was implemented by querying the virtual private network (VPN) travel and contact history database maintained by the Ministry of Health and Welfare (Figure 1). Patients are required to enter their national ID number when attempting to book an appointment (Figure 2). The system then uses the national ID number to check for relevant travel or contact history through the VPN database. If the result turns out to be negative, an appointment is booked, and patients who entered their mobile phone numbers will receive text messages that provide them with access to an expedited entry route. However, if a patient has a potentially problematic travel or contact history, the attempt to book an appointment will fail. Patients can also book appointments on site. Their history will then be checked via the VPN database on inserting their health smart card before gaining entry through the regular route.

Upon arrival at the hospital, outpatients can enter the hospital through two routes: regular or expedited. The regular route is for patients who do not present text messages received from the screening system. These patients are asked to present their health smart card to the staff. The on-site VPN database query is then made, and a patient would be granted access after the confirmation of no potentially problematic histories provided by the VPN database. The patient will also receive a paper-based questionnaire that is to be submitted to the hospital in the examination room.

On the other hand, patients who successfully book an appointment on the internet and receive text messages from the hospital can enter the hospital through the expedited route. The staff will confirm the legitimacy of the text message and verify the patient’s identity with the patient’s health smart card. Once verified, the patient is allowed to enter the hospital and is provided the same aforementioned questionnaire.

### Data Processing

The system described in this article was deployed on April 21, 2020. Data were retrieved from the registration database between April 21 and May 10, 2020. The data for each Sunday during that period were omitted since no regular outpatient service is provided on Sundays. All web-based and on-site booked appointments were extracted. Each denied appointment was also extracted along with the reason for nonadmittance during the studied period.

### Statistical Analysis

Descriptive statistics were assessed using Microsoft Excel 2016 (Microsoft Corp).

## Results

During the study period, 127,857 visits were recorded. Among these visits, 91,195 were registered on the internet. In total, 71,816 of them generated text messages for the expedited route of entry (Table 1).

The average daily number of visits during the study period was 9498 on weekdays and 1463 on Saturdays. The highest number of visits (n=11,147) was recorded on May 6, 2020, and the lowest number (n=1351) was recorded on April 25, 2020.

In total, 71% of the visits were registered on the internet. Further, 78.76% of all patients who registered on the internet received text messages delivered from the system for the expedited route of entry.

Of all the visits registered on the internet, queries to the VPN database revealed relevant histories for 65 patients. Moreover, 66 patients entered invalid national ID numbers when attempting to register for an outpatient visit. All of these attempts were blocked by the system. The denied attempts comprised approximately 0.14% of all web-based registration attempts.

**Table 1:** Total number of attempts and visits from April 21 to May 9, 2020.

Observations	April 21	April 22	April 23	April 24	April 25	April 27	April 28	April 29	May 1	May 2	May 4	May 5	May 6	May 7	May 8	May 9	Total
Attempts with an invalid ID, n	6	7	5	3	0	7	5	4	2	0	7	5	6	4	5	0	66
Attempts with relevant history, n	6	7	4	3	1	11	3	5	3	2	5	2	3	5	3	2	65
Text messages sent, n	4121	4961	5083	4434	848	5330	5358	6051	4693	908	5728	5699	6550	5816	5204	1032	71,816
Attempts to register, n	5468	6660	6363	5610	1010	6828	6781	7679	5960	1091	7171	7243	8163	7340	6622	1206	91,195
Total outpatient visits, n	8154	9294	8910	7866	1351	10,071	9247	10,405	8309	1437	10,650	10,017	11,147	10,128	9270	1601	127,857
Web-based registration rate of outpatients, %	67.06	71.66	71.41	71.32	74.76	67.80	73.33	73.80	71.73	75.92	67.33	72.31	73.23	72.47	71.43	75.33	71.33
Text message receipt rate on web-based attempts, %	75.37	74.49	79.88	79.04	83.96	78.06	79.01	78.80	78.74	83.23	79.88	78.68	80.24	79.24	78.59	85.57	78.75

## Discussion

### Principal Findings

To our knowledge, this is the first reported system to automatically screen for a patient’s travel and contact histories prior to entry to a hospital. A similar check-in system for outpatient magnetic resonance imaging studies has been previously described [10], but there have been no reports of a hospital-wide service for the extraction of travel and contact histories. Previous studies on web-based outpatient resources of hospitals have focused on probing the features of official applications and appointment systems [11,12]. The purpose of the system reported herein is to deny access to patients with a relevant contact or travel history recorded in the VPN contact and travel history database. The goal was successfully achieved after the system’s deployment on April 21, 2020.

### Challenges of Screening a Massive Number of Outpatients

Taipei Veterans General Hospital, being one of the largest public academic centers in Taiwan, is visited by over 10,000 patients each day. The traditional method of screening outpatients for relevant travel, occupation, cluster, and contact histories involves medical professionals or administrative staff taking patient histories and excluding those with relevant histories. This traditional method has allowed patients with relevant travel and contact histories to enter the hospital and could thus facilitate further disease spread. Therefore, a system to deny entry to patients with relevant histories was warranted [5].

However, with the large number of patients visiting the hospital’s outpatient department, an efficient way to acquire their histories was also required. Therefore, the automatic screening system utilizing the VPN travel and contact history database was an optimal solution for the obstacles encountered. The system acquires these histories by querying the database with the provided national ID numbers. With minimal training, staff could easily utilize the on-site screening system at the hospital entrance to stop patients with relevant histories from entering the outpatient department.

### Long Lines in the Early Phase of the Screening Policy

During the early phase of implementing the screening policy for outpatients, the hospital relied on on-site VPN database queries to deny entrance to patients with a relevant travel or contact history reported by the database. However, this soon proved time-consuming. Long lines were formed each morning during the opening hours of ambulatory services. Therefore, an expedited entry route was further designed to alleviate this problem.

The expedited route provided a faster track to patients with registered mobile phone numbers that passed the VPN database check before arriving at the hospital and was able to successfully mitigate the long lines at the entry checkpoint. Additionally, denying patients with a relevant history upon booking an appointment on the internet stops such patients from arriving at the hospital and further lowers the risk of disease spread.

### The Need for a Paper-Based Questionnaire

Though querying the VPN travel and contact database provided a fast and efficient means to deny hospital entry to individuals with a relevant travel or contact history, the information provided by the database was incomplete. Therefore, a separate questionnaire was needed for recording each patient’s occupational history and other pertinent information.

To ensure that every person visiting the outpatient department completed this questionnaire, a paper-based version of the questionnaire was distributed to each patient at the hospital entrance and then collected upon the patient’s entry to the examination room before the patient was examined by the physician.

### Presence of Contact and Travel Histories Among Outpatients

The system revealed a total of 65 patients with relevant histories, including those with recent travel histories to high-risk countries within 2 weeks or those with positive contact histories, during the study period. This accounted for an extremely low percentage (0.05% of total visits during the study period) of the entire outpatient population.

However, one case of a nosocomial cluster at a medical center in Taiwan still indicated the importance of enforcing rigorous entrance screening policies at large medical facilities.

### Limitations

This study aimed to illustrate the design and utilization of an automatic system to acquire patients’ relevant travel and contact histories. However, owing to the limited time spent on its development, the system was unable to generate records in the electronic medical record system. Moreover, the on-site screening system could not distinguish on-site–registered outpatients from family members and friends accompanying the patients. Therefore, no analysis could be performed for individuals who registered on site.

### Conclusions

This study demonstrated the successful deployment of an automatic system to acquire patients’ relevant travel and contact histories. The utilization rate of the internet-based system is optimal (78.76% of all web-based registered visits) since it is incorporated into the already operating web-based registration system for outpatient visits. Further efforts could be made to integrate the system with the electronic medical record system.

## Abbreviations

VPN: virtual private network
